# Coping with stress: mechanics of the expanding leaf

**DOI:** 10.1093/jxb/erw340

**Published:** 2016-10-04

**Authors:** Anne-Lise Routier-Kierzkowska, Daniel Kierzkowski

**Affiliations:** Department of Comparative Development and Genetics, Max Planck Institute for Plant Breeding Research, Carl-von-Linné-Weg 10, 50829 Cologne, Germany

**Keywords:** Elasticity, growth, leaf, mechanical measurements, strain-stiffening.

**The precise control of physical properties of growing tissues is crucial for plant morphogenesis. Sahaf and Sharon (pages 5509–5515 in this issue) examined the mechanics of the expanding leaf and showed that plant tissues respond to stress by changing their mechanical properties. A new method is proposed to distinguish reversible and irreversible tissue deformation, an important step in understanding the physics of a growing cell wall. Leaf blades could hold the key to understanding how plants regulate their growth in different directions.**

In recent years, considerable progress has been made in research on the molecular and genetic basis of plant morphogenesis. However, organ growth is governed by physical processes occurring at multiple scales. Plant cells grow due to the irreversible (or *plastic*) deformation of their stiff cell wall under tension. The cell wall is a complex hydrated gel composed mainly of pectins and hemicellulose reinforced by stiff cellulose microfibrils. Different models of plant cell wall structure agree on the load-bearing role of cellulose, but the mechanical function of other wall components remains unclear (Cosgrove, 2015). Precise measurements of cell wall behavior under various mechanical conditions are needed to further refine and validate structural models.

The mechanical force driving expansion of plant cells primarily results from turgor pressure. In an isolated cell, the magnitude and orientation of tensile stresses is determined only by its geometry and internal pressure. In an expanding tissue, local differences in wall properties or stresses would in theory cause individual cells to grow at different rates. Plant cells are, however, glued to their neighbors via cell walls, forcing them to grow as a continuous tissue and creating residual stresses, also termed *tissue stresses* (Baskin and Jensen, 2013). In modeling terms, this means the *specified growth* (i.e. the expansion cells would exhibit if they did not have neighbors) differs from the *resultant growth*, i.e. the only expansion we can actually observe (Kennaway *et al.*, 2011). The discrepancy between specified and resultant growth depends on how tissues deal with residual stresses, e.g. reducing stresses by deforming passively or building stresses up by resisting them. Since neither specified growth nor mechanical stresses can be measured directly, the best way to investigate this reaction is to apply an external force to a growing tissue.

Sahaf and Sharon (2016) show that tobacco leaves expand globally at a similar rate in all directions under natural conditions, i.e. tissue growth is isotropic. However, the leaf blade reacts to additional mechanical stress in a very remarkable way (Box 1). When subjected to a constant force for a short period of time, the tissue elongates in the direction of imposed stretch and shrinks laterally due to the Poisson effect, much as a piece of rubber would do. Over longer time scales an active response becomes visible, which causes the elongation rate in the stretched direction to slow down to the same values as in the unstretched part of the leaf and for growth to be normal in the opposite direction despite the Poisson effect. In short, the leaf blade appears to fight against external mechanical stresses, maintaining its specified growth in both directions.

**Box 1. Growth adapts to external mechanical constraints and correlates with tissue elasticity**

Applying a constant force (black arrows) results in fast tissue stretching (white bar) in the direction of applied load and tissue contraction (red bar) in the perpendicular orientation due to the Poisson effect (A). Growth under constant load is first oriented along the applied force (B) and then becomes isotropic (C) as in the non-stretched side (left). Releasing load causes growth reorientation, which becomes perpendicular to the previously applied force (D) and turns back to isotropy only after several hours (E). Before loading, leaf tissue is equally elastic in both orientations. Prolonged load leads to tissue stiffening parallel to, and softening perpendicular to, the applied force (F).

**Figure:**
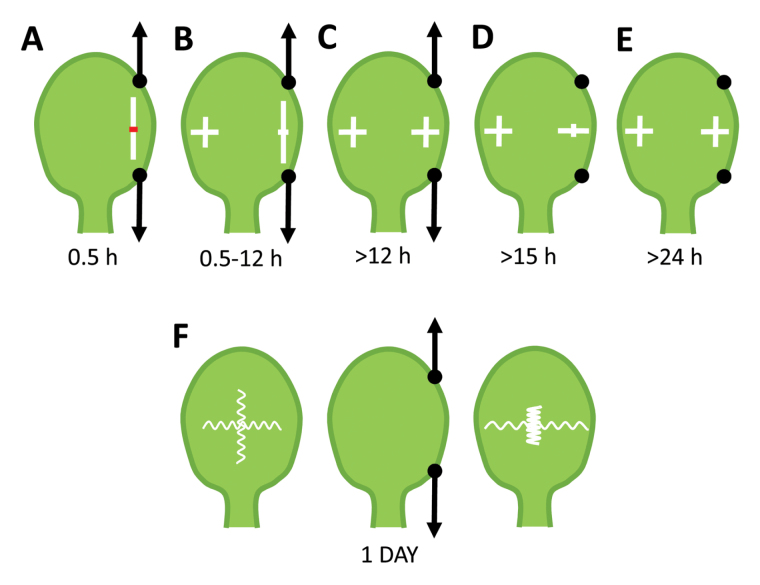

## Plastic and elastic growth

What changes in mechanical properties underlie this coping mechanism? A possible candidate would be modifications in elasticity (reversible deformation of the tissue). The link between elasticity and growth is still unclear and even controversial (Cosgrove, 2015), although it has been demonstrated in different studies (reviewed by Routier-Kierzkowska and Smith, 2013). Part of the problem in measuring the reversible component of deformation in a growing organ is that tissues are often visco-elastic, i.e. they take some time to return to their original configuration (Nolte and Schopfer, 1997). To understand the relationship between plastic and visco-elastic deformations Sahaf and Sharon used the same experimental setup in two different ways. While they applied a constant force over long periods of time to mimic growth stresses, visco-elasticity was assessed by measuring tissue deformation under an oscillating load (Box 2).

**Box 2. Measuring reversible vs plastic properties**

A static load (red curve) can be used over long periods of time to mimic the effects of tissue stresses and study the plastic deformation (green curve) of the leaf blade (A). At first the tissue exhibits a fast linear deformation that is mainly reversible (vertical segment of green curve) and later slowly creeps (plastic or irreversible deformation) until reaching a steady growth rate. On a smaller timescale, visco-elasticity is measured by applying an oscillating force (red curve) to the tissue (B). Loading cycles occur sufficiently fast such that creep does not take place and the amplitude of deformation (green curve) stays constant. The curve’s slope reflects tissue elasticity, while the time shift between curves indicates viscous relaxation. The technique used by Sharon and Sahaf probes tissue elasticity by adding mechanical stress to those already existing. Measuring cell deformation upon osmotic treatments, on the other hand, gives an insight into cellular elasticity at different levels of turgor-based mechanical stress (Kierzkowski *et al.*, 2012), as shown here in the shoot apical meristem (C). Combining both approaches would provide an exciting new perspective on cell wall modifications due to stress.

**Figure:**
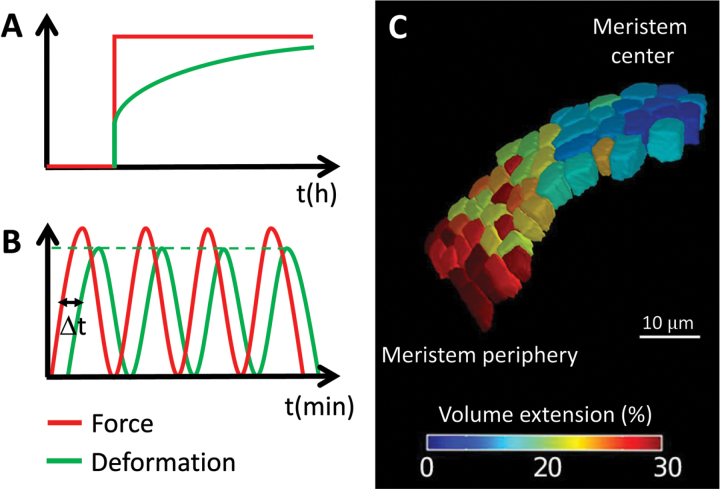

Sahaf and Sharon show that in the leaf blade changes in plastic behavior induced by stress do correlate with modifications in visco-elastic properties. They observe a clear increase in stiffness along the direction of imposed stretch. This behavior is in accordance with previous findings that cortical microtubules orient according to the direction of maximal stress, guiding the deposition of new cellulose microfibrils (Hejnowicz *et al.*, 2000; Hamant *et al.*, 2008). Since microfibrils determine the direction in which the cell wall is stiffer both in term of plastic and visco-elastic deformations (Baskin and Jensen, 2013; Cosgrove, 2015), the alignment of newly deposited cellulose fibres is a likely candidate for tissue stiffening in the direction of imposed stress. More surprisingly, the tissues actually become elastically softer in the opposite direction. In this case visco-elasticity also correlates with plastic behavior. Since the tension in this direction was reduced due to the initial Poisson effect, normal lateral growth rates can be interpreted as the effect of a higher plastic compliance. This simultaneous softening and stiffening in opposite directions has not been shown before, possibly because most studies of tissue elasticity have been conducted on long and thin samples, such as young stems, which only allow measurements along one axis. Paradoxically, leaves which normally do not exhibit a preferential growth axis could provide new model systems to elucidate how mechanical properties are regulated in different directions.

## Back to the cell wall

Several scenarios explaining blade softening in the direction perpendicular to applied stress could be investigated. The primary cell wall exhibits a polylamelate structure in which cellulose microfibril orientation in-between successive layers can vary abruptly (Zhang *et al.*, 2016). Stress is distributed unevenly across wall layers and it is possible that different layers are responsible for bearing transverse and longitudinal stress (Hejnowicz and Borowska-Wykret, 2005). It is conceivable that selective softening of older layers, in parallel with the deposition of a new layer reinforced in the direction of applied stress, could explain the opposite regulation of tissue stiffness in both directions. This could be investigated by using Atomic-force or Scanning Electron microscopy to probe the modifications in nano-structure of cell wall layers (Zhang *et al.*, 2016) induced by constant load.

Leaves also provide an interesting system for exploring subcellular mechanical regulation of growth. In Arabidopsis, despite a relatively uniform expansion of leaves at the tissue scale (Remmler and Rolland-Lagan, 2012), puzzle-shaped epidermal cells often display sharp local growth differences at the cell wall level (Elsner *et al.*, 2012; Armour *et al.*, 2015). The complex shape of epidermal leaf cells also results in non-trivial stress patterns (Sampathkumar *et al.*, 2014). One could imagine that, within puzzle-shaped cells, the orientation of individual lobes with respect to the applied tensile stress could determine the local softening or stiffening of the cell wall. Such local modifications could induce changes in mechanical anisotropy at the global scale. Testing such a possibility would require leaf growth to be followed at very high resolution, for example using confocal microscopy (Vlad *et al.*, 2014), and combine stretching experiments with tracking of subcellular deformations. Cellular and subcellular elasticity could also be assessed using turgor manipulation (reviewed by Cosgrove, 2015), a technique recently used to assess anisotropic elastic properties of single cells (Weber *et al.*, 2015; Hofhuis *et al.*, 2016) as well as non-linear elasticity (Kierzkowski *et al.*, 2012).

Despite renewed interest in plant mechanics, fundamental questions regarding growth regulation still need to be answered. Because they cannot be observed directly, the different kinds of mechanical stresses driving cell expansion have often been omitted, leading to apparent discrepancies between the observed growth and cell wall structure (Baskin and Jensen, 2013). Using the leaf as a new model could help bridge the gap between wall micro-mechanics, tissue stresses and growth.
